# Correction: CKLF1 Aggravates Focal Cerebral Ischemia Injury at Early Stage Partly by Modulating Microglia/Macrophage Toward M1 Polarization Through CCR4

**DOI:** 10.1007/s10571-024-01480-7

**Published:** 2024-05-10

**Authors:** Chen Chen, Shi‑Feng Chu, Qi‑Di Ai, Zhao Zhang, Fei‑Fei Guan, Sha‑Sha Wang, Yi‑Xiao Dong, Jie Zhu, Wen‑Xuan Jian, Nai‑Hong Chen

**Affiliations:** 1 State Key Laboratory of Bioactive Substances and Functions of Natural Medicines, Institute of Materia Medica & Neuroscience Center, Chinese Academy of Medical Sciences and Peking Union Medical College, Beijing, 100050 China; 2 Hunan Engineering Technology Center of Standardization and Function of Chinese Herbal Decoction Pieces & Hunan University of Chinese Medicine First-Class Disciple Construction Project of Chinese Materia Medica, Changsha, 410208 China; 3 Key Laboratory of Human Disease Comparative Medicine, Institute of Laboratory Animal Science, NHFPC, Peking Union Medicine College and Chinese Academy of Medical Sciences, Beijing, 100021 China; 4 School of Basic Medicine, Shanxi University of Traditional Chinese Medicine, Taiyuan, 030619 China; 5 Tianjin University of Traditional Chinese Medicine, Tianjin, 300193 China; 6 Institute of Process Engineering, Chinese Academy of Sciences, Beijing, 100050 China; 7 DME Center, Clinical Pharmacology Institute, Guangzhou University of Chinese Medicine, Guangzhou, 510000 China

**Correction to** **:** **Cellular and Molecular Neurobiology (2019) 39(5):651–669** 10.1007/s10571-019-00669-5

The original version of this article unfortunately contained error in Fig. 10.

**Fig. 10:**
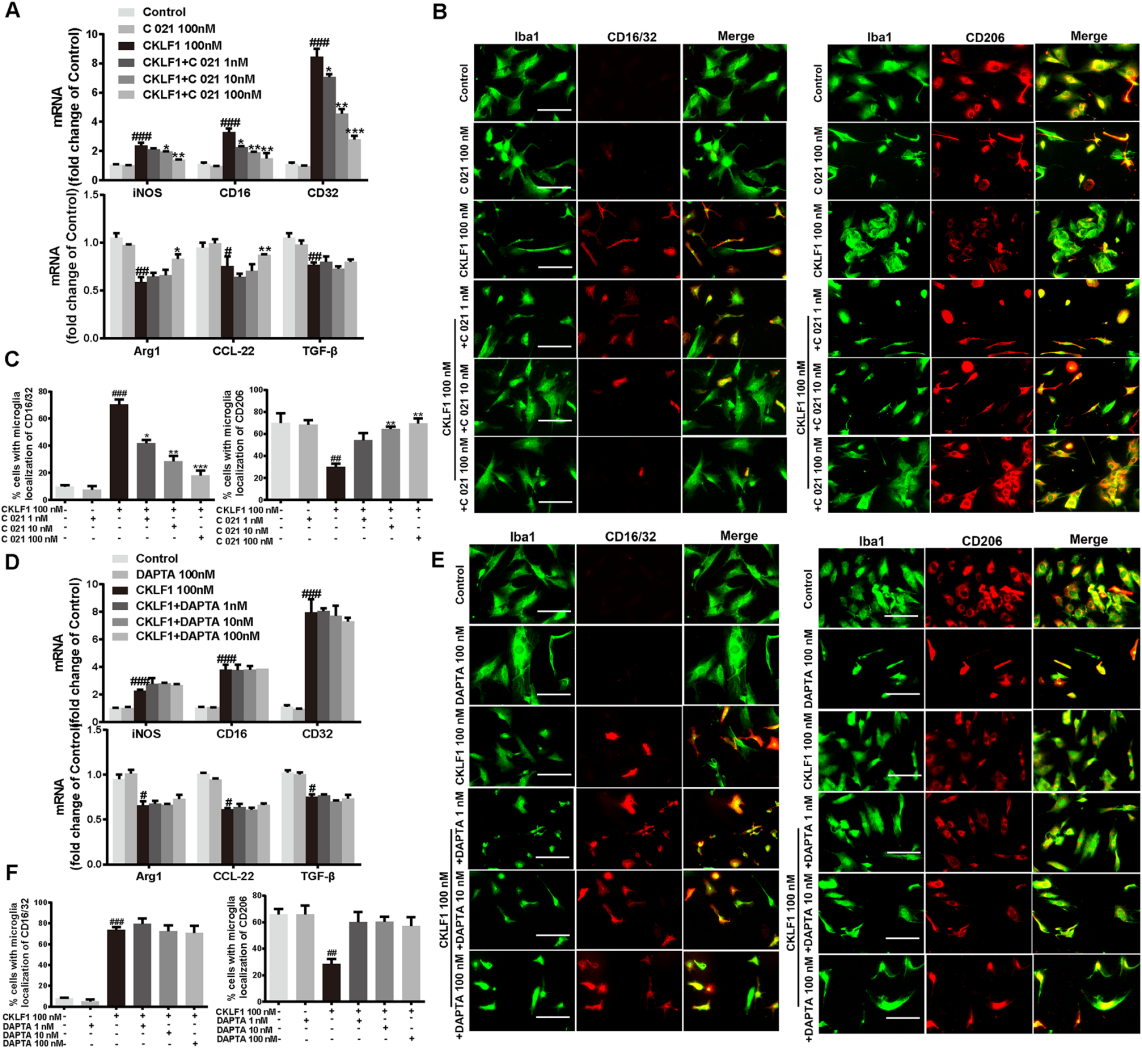
CKLF1 modulates the microglia polarization through CCR4. **a** qPCR analysis of mRNA expression levels of M1 markers (iNOS, CD16, CD32) and M2 markers (Arg1, CCL-22, TGF-β) in primary microglia treated with CKLF1 and C 021 dihydrochloride for 24 h (*n* = 6 cell samples). ^#^*P* < 0.05, ^##^*P* < 0.01, ^###^*P* < 0.001 versus control; *P < 0.05, **P < 0.01, ***P < 0.001 versus CKLF1 100 nM. **b** Representative photomicrographs of double-staining immunofluorescence of CD16/32 or CD206 with Iba1 in the primary microglia treated with CKLF1 and C 021 dihydrochloride for 24 h. Scale bars 100 μm. **c** Quantitative analysis of CD16/32-positive and CD206- positive microglia (*n* = 6 cell samples). ^##^*P* < 0.01, ^###^*P* < 0.001 versus control; *P < 0.05, **P < 0.01, ***P < 0.001 versus CKLF1 100 nM. **d** qPCR analysis of mRNA expression levels of M1 markers (iNOS, CD16, CD32) and M2 markers (Arg1, CCL-22, TGF-β) in primary microglia after treatment with CKLF1 and DAPTA for 24 h (*n* = 6 cell samples). ^#^*P* < 0.05, ^###^*P* < 0.001 versus control. **e** Representative photomicrographs of double-staining immunofluorescence of CD16/32 or CD206 with Iba1 in the primary microglia after treatment with CKLF1 and DAPTA for 24 h. Scale bars 100 μm. **f** Quantitative analysis of CD16/32-positive and CD206-positive microglia (*n* = 6 cell samples). ^##^*P* < 0.01, ^###^*P* < 0.001 versus control

In Fig. 10B, the merge image of C021 100 nM group of microglia stained with CD206 is published incorrectly. Other images in the figure remains the same, and the interpretation of the results remains unchanged.

The corrected figure is presented here.

The authors would like to apologise for any inconvenience caused.

The original article has been corrected.

